# A Rare Case of Rosai-Dorfman Disease Involving Great Vessels of the Heart Mimicking Breast Cancer Recurrence

**DOI:** 10.1016/j.atssr.2026.03.010

**Published:** 2026-04-01

**Authors:** Nour Chres, Giovanni Mattioni, Luca Pogliani, Eleonora Poirè, Marco Reda, Giacomo Grisorio, Serena Conforti, Arash Astaneh

**Affiliations:** 1 Department of Thoracic Surgery, ASST Grande Ospedale Metropolitano Niguarda, Milan, Italy; 2 School of Thoracic Surgery, University of Milan, Milan, Italy

## Abstract

Rosai-Dorfman disease (RDD) is a rare non-Langerhans histiocytosis, with cardiovascular involvement in <0.1% of cases. We report the case of a 69-year-old woman with history of breast cancer found to have mediastinal nodules involving the superior vena cava and pulmonary veins. Imaging suggested recurrence of malignant disease, and multiple biopsies were nondiagnostic. Definitive diagnosis was achieved after median sternotomy under cardiopulmonary bypass with superior vena cava resection and prosthetic reconstruction. Histopathologic analysis confirmed RDD with abundant IgG4-positive plasma cells. This case illustrates the diagnostic complexity of extranodal RDD and the role of surgery in cardiovascular involvement.

Rosai-Dorfman disease (RDD) is a rare non-Langerhans cell histiocytosis that can involve both nodal and extranodal sites, although extranodal involvement is uncommon.^1^ Cardiovascular involvement is exceedingly rare. We present a challenging case of RDD with involvement of the superior vena cava (SVC) and pulmonary veins.

A 69-year-old woman with a history of left breast cancer, treated with surgery and adjuvant hormone therapy, underwent a routine 6-month follow-up computed tomography (CT) scan. The scan revealed 3 solid, rounded nodules: 1 in the subcarinal region, 1 at the lower margin of the left inferior pulmonary vein, and 1 lateral to the SVC (Figure 1A). The patient was asymptomatic, with an unremarkable physical examination and normal vital signs. Subsequent ^18^F-fluorodeoxyglucose positron emission tomography/CT demonstrated hypermetabolism (maximal standardized uptake value of 7.3) in the subcarinal and inferior pulmonary vein nodules but not in the nodule adjacent to the SVC (Figure 1B). These findings were initially interpreted as possible lymphadenopathies. Considering the patient’s oncologic history, the multidisciplinary tumor board discussed a differential diagnosis that included metastatic lymph node recurrence of breast carcinoma vs new-onset sarcoidosis. A diagnosis was therefore mandatory. Endobronchial and endoscopic ultrasound-guided needle aspiration biopsies were performed, but both yielded negative results. Consequently, 5 months later, a surgical biopsy of the subcarinal lymph node was performed through triportal video-assisted thoracic surgery. Histologic analysis was negative for malignancy and demonstrated diffuse sinus histiocytosis. On the basis of these findings, after multiple failing diagnostic attempts, the multidisciplinary tumor board proposed radiologic follow-up. The new imaging 1 year after revealed progression in lesion size, with the SVC nodule now showing hypermetabolic activity (maximal standardized uptake value of 9.77; Figures 1C, 1D, 1E). Given the concerning evolution and the previous nondiagnostic surgical biopsy, excision of the right-sided nodule adjacent to the SVC was planned to obtain a diagnosis. Although lymphadenopathy remained the main suspicion, a careful review of imaging raised the possibility of an intrapericardial solid mass rather than a lymph node. Given the diagnostic uncertainty, we first opted for a less invasive exploratory approach. Three months later, the patient therefore underwent a right lateral muscle-sparing thoracotomy with hilar and mediastinal exploration. On opening of the pericardium, a firm, fibrous, grayish mass was identified, embedded in the wall of the SVC near the cavoatrial junction. Because of suboptimal exposure for safe resection or biopsy, the procedure was concluded, and a definitive diagnostic resection was planned under cardiopulmonary bypass through median sternotomy. As part of the preoperative workup, transthoracic echocardiography and coronary angiography were performed, both showing no abnormalities.

**Figure 1:**
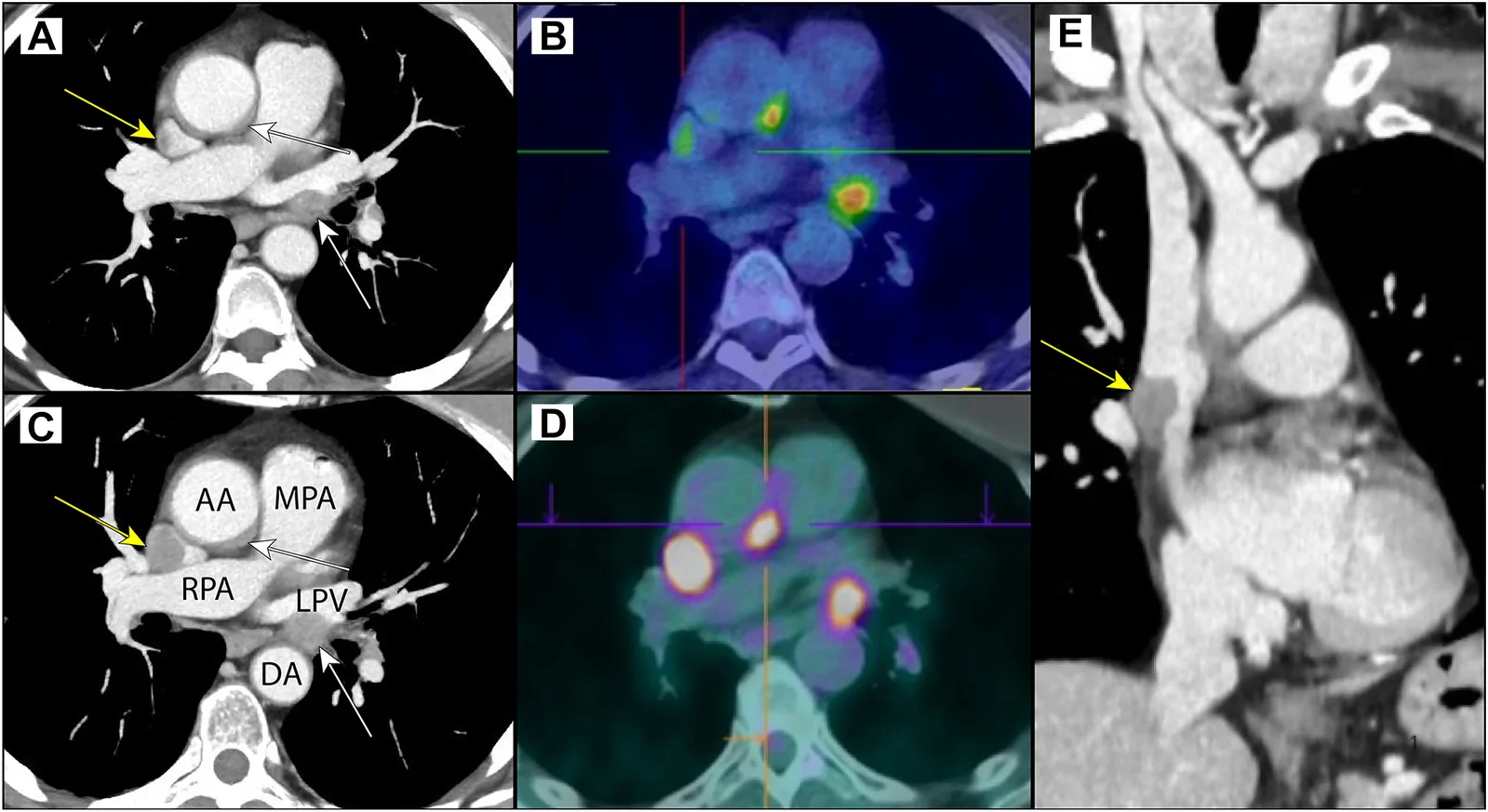
Computed tomography (CT) and ^18^F-fluorodeoxyglucose positron emission tomography (^18^F-FDG PET) images. (A) Baseline axial contrast-enhanced CT scan showing the nodule within the wall of the superior vena cava (yellow arrow) and additional nodules in the left atrial wall and at the lower margin of the left inferior pulmonary vein (white arrows). (B) Baseline ^18^F-FDG PET scan demonstrating initial modest hypermetabolic activity of the nodule. (C) Follow-up axial contrast-enhanced CT scan revealing increased size of the nodules (yellow arrow). Again, the white arrows show additional nodules in the left atrial wall and at the lower margin of the left inferior pulmonary vein. (AA, ascending aorta; DA, descending aorta; LPV, left pulmonary vein; MPA, main pulmonary artery; RPA, right pulmonary artery.) (D) Follow-up ^18^F-FDG PET scan showing marked increased metabolic activity. (E) Coronal CT scan depicting invasion of the superior vena cava by the nodule (yellow arrow).

The patient subsequently underwent median sternotomy under cardiopulmonary bypass, established with central aortic and bicaval cannulation. The SVC was cannulated cranially to the nodule. Intraoperatively, 2 additional nodules were discovered: one within the left atrial wall and another arising from the ostium of the left inferior pulmonary vein, extending toward the hilum. The SVC nodule together with the involved vascular segment was resected and reconstructed with a 16-mm expanded polytetrafluoroethylene (exGraft; PECA Labs) straight vascular prosthesis (Figures 2A, 2B). The intraoperative frozen section examination considered the specimen to be suggestive of a possible mesenchymal origin disease. The other 2 nodules were left in place, given the absence of a definitive diagnosis at that time, and a resection was considered too risky without a clear therapeutic strategy.

**Figure 2:**
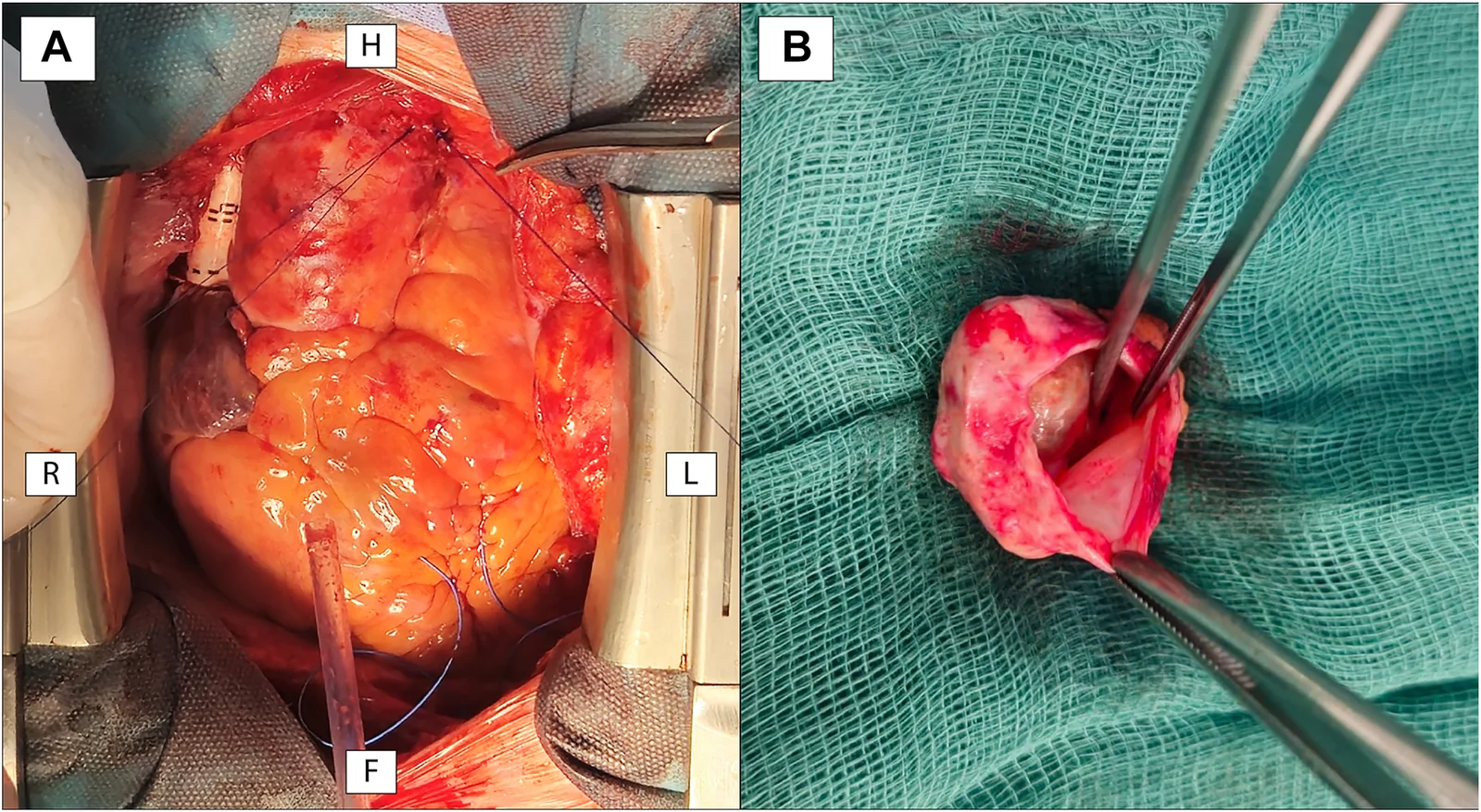
Intraoperative images. (A) In median sternotomy after decannulation, the image shows the straight vascular prosthesis of the superior vena cava (expanded polytetrafluoroethylene exGraft 16 mm). (F, feet; H, head; L, left; R, right). (B) Resected specimen of superior vena cava including Rosai-Dorfman nodule, which appears as a firm, grayish nodule.

The postoperative course was uneventful. The patient was discharged on postoperative day 8.

Final histopathologic analysis revealed a 2 × 2-cm grayish nodule composed of prominent sinus histiocytosis with abundant IgG4-positive plasma cells, consistent with RDD. The immunohistochemical profile demonstrated positivity for Oct-2, CD68, CD163, S100, and cyclin D1 while being negative for CD1a, SOX-10, ALK1, GATA-3, synaptophysin, desmin, GFAP, CK AE1/AE3, and actin. The patient was referred to a rheumatologist for further evaluation and management. At 6 months after surgery, she remains asymptomatic and in good clinical condition, with stable unresected nodules on radiologic follow-up (Figure 3). No medical treatment has been initiated because the patient is currently asymptomatic and the RDD lesions are radiologically stable. A summary of the patient’s timeline is reported in the Supplemental Figure.

**Figure 3:**
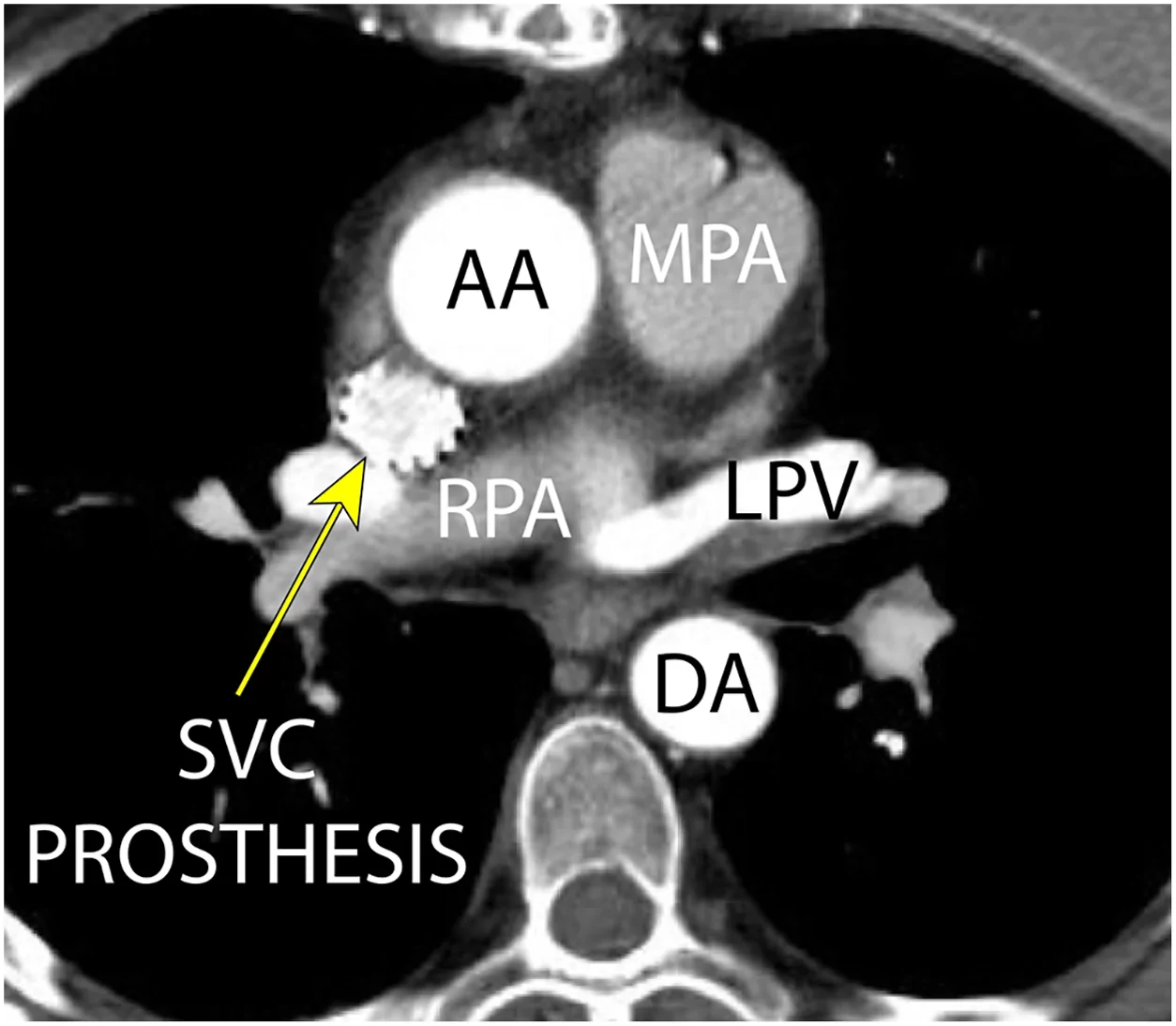
Follow-up imaging. Contrast-enhanced axial computed tomography scan 6 months after surgery showing the prosthesis of the superior vena cava (SVC) (yellow arrow) and the 2 Rosai-Dorfman disease lesions that are morphologically stable. (AA, ascending aorta; DA, descending aorta; LPV, left pulmonary vein; MPA, main pulmonary artery; RPA, right pulmonary artery.)

## Comment

This case illustrates the diagnostic challenges of RDD, particularly in a patient with a history of malignant disease. RDD is typically manifested as painless cervical lymphadenopathy, but extranodal involvement occurs in a minority of cases. Common extranodal sites include the skin, soft tissues, upper respiratory tract, bone, and central nervous system; cardiovascular involvement is extremely rare, occurring in <0.1% of cases.^1^ The wide clinical spectrum necessitates a broad differential diagnosis.^2^ To date, only a few cases of vascular involvement have been reported in the literature; to our knowledge, involvement of the SVC has not yet been described. Further details concerning the other cases of vascular involvement are briefly reported in the Supplemental Material.

On CT scans, RDD usually appears as a solid, homogeneously enhancing nodule. Positron emission tomography imaging often demonstrates intense ^18^F-fluorodeoxyglucose uptake,^3^ although in our patient this was not initially the case, complicating the diagnostic process. Magnetic resonance imaging can further assist in differential diagnosis as RDD lesions typically appear isointense to hypointense on T1- and T2-weighted sequences, with diffuse, homogeneous contrast enhancement.^4^

Given the nonspecific imaging features, definitive diagnosis relies on histopathologic evaluation. Hallmark findings include a proliferation of histiocytes exhibiting emperipolesis along with inflammatory infiltration. Immunohistochemistry is typically positive for CD68, CD163, and S100 and negative for CD1a and langerin.^5^

RDD is generally benign, and treatment strategies depend on disease location, extent, and symptoms. Systemic disease may respond to corticosteroids, immunosuppressants, or chemotherapy. In contrast, when vital structures are affected, the disease can become life-threatening, making surgical resection the treatment of choice in localized presentations.

## Figures and Tables

**Supplementary Figure 1:**
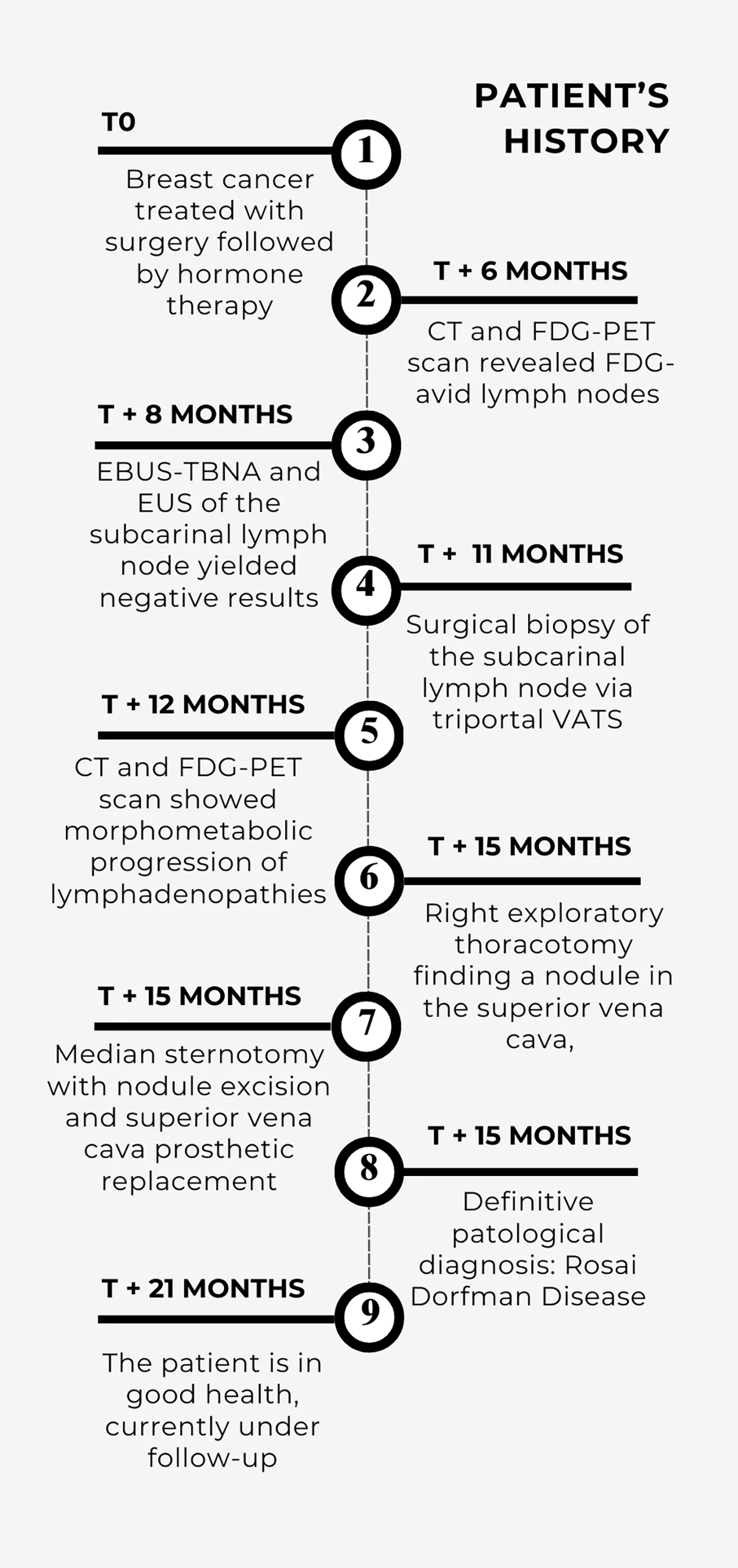


## Supplementary Data

Supplementary Figure 1 caption
