# Supplementary material for: A Rare Case of Rosai-Dorfman Disease Involving Great Vessels of the Heart Mimicking Breast Cancer Recurrence
Source: Ann Thorac Surg Short Rep. 2026 Apr 1;4(3):1000–3. doi: 10.1016/j.atssr.2026.03.010 (PMC13555473; doi:10.1016/j.atssr.2026.03.010)
Supplement: Supplementary file 1 [file mmc1.docx]

**FIGURE LEGEND**

Figure S1. **Patient’s timeline**.

Illustration of patient’s disease history timeline. EBUS-TBNA = Endobronchial Ultrasound-guided Transbronchial Needle Aspiration; EUS = Endoscopic Ultrasound; VATS = Video-Assisted Thoracic Surgery
