# Supplementary material for: A Rare Case of Rosai-Dorfman Disease Involving Great Vessels of the Heart Mimicking Breast Cancer Recurrence
Source: Ann Thorac Surg Short Rep. 2026 Apr 1;4(3):1000–3. doi: 10.1016/j.atssr.2026.03.010 (PMC13555473; doi:10.1016/j.atssr.2026.03.010)
Supplement: Supplementary file 2 [file mmc2.docx]

Supplementary Text T1.

**List of studies reporting Rosai Dorfman disease with cardiovascular involvement on PubMed Advanced Search Builder.**

A review of the literature identified eight published cases of Rosai–Dorfman disease (RDD) with cardiovascular involvement. The following text summarizes each case, including the affected cardiovascular structures, clinical presentation, surgical management and follow-up when reported.

The first case, described by **Walters et al. (2010)**, involved a **22-year-old woman** presenting with **right heart failure** due to a mass affecting the **main pulmonary artery** and the **origins of both pulmonary arteries**. She underwent **arteriotomy and debulking** of the lesion, with an uneventful postoperative course and **5-month follow-up**.

In **2013**, **Morsolini et al.** reported a **62-year-old man** with isolated involvement of the **right pulmonary artery**. No symptoms were documented. He underwent endoarterectomy of the **right pulmonary artery**, with **9-month follow-up** showing stability.

**Guozhu et al. (2020)** described a **48-year-old woman** presenting with **shortness of breath**, found to have RDD involving the **pulmonary artery**. She underwent **pulmonary endoarterectomy**, with **3-month follow-up** reported.

A similar case was reported by **Liu et al. (2021)**: a **48-year-old woman** with **dyspnea** and a lesion affecting the **main pulmonary artery** and the **origins of both left and right pulmonary arteries**. The patient underwent **bilateral pulmonary artery resection and reconstruction**, with **8-month follow-up**.

In **2023**, **Li et al.** presented a **41-year-old woman** with RDD of the **descending aorta**, who complained of **hypertension and dizziness**. She was treated with **descending aortic prothesis replacement** and she underwent 6 months follow-up with clinical stability.

**Dronamraju et al. (2023)** reported a **33-year-old woman** with **dyspnea**, who had a mass involving the **main pulmonary artery**. Surgical **excision of the mass** was performed, and the patient underwent **12 weeks follow-up**.

In **2024**, **Nguyen et al.** described a **63-year-old woman** with an extensive mass originating from the **left atrium** and **interatrial septum**, displacing the **right atrium** and causing **obstruction at the junction of the right atrium and inferior vena cava**. She presented with **chest pain and atrial flutter** and underwent **cavo-atrial bypass**. **Follow-up data were not reported**.

Finally, **Sergio et al. (2024)** reported a **54-year-old woman** with involvement of the **right atrium and tricuspid valve**, presenting with **chest pain**. She underwent **excision of the right atrial mass**, with **1-year follow-up** demonstrating good postoperative outcome.
